# Relatives Experience More Psychological Distress Due to COVID-19 Pandemic-Related Visitation Restrictions Than In-Patients

**DOI:** 10.3389/fpubh.2022.862978

**Published:** 2022-07-13

**Authors:** Sabine Felser, Corinna Sewtz, Ursula Kriesen, Brigitte Kragl, Till Hamann, Felix Bock, Daniel Fabian Strüder, Clemens Schafmayer, Désirée-Louise Dräger, Christian Junghanss

**Affiliations:** ^1^Hematology, Oncology, Palliative Medicine, Department of Internal Medicine, Clinic III, University Hospital Rostock, Rostock, Germany; ^2^Department of Neurology, University Hospital Rostock, Rostock, Germany; ^3^Department of Radiation Oncology, University Hospital Rostock, Rostock, Germany; ^4^Head and Neck Surgery “Otto Koerner”, Department of Otorhinolaryngology, University Hospital Rostock, Rostock, Germany; ^5^Department of General Surgery, University Hospital Rostock, Rostock, Germany; ^6^Department of Urology, University Hospital Rostock, Rostock, Germany

**Keywords:** communication, COVID-19, psychological distress, stress, visit restriction

## Abstract

**Background:**

The COVID-19 pandemic led to visiting restrictions (VRs) of patients in hospitals. Social contacts between patients' relatives play an important role in convalescence. Isolation may cause new psychological comorbidity. The present study investigated the psychological distress of VR in in-patients and their relatives.

**Methods:**

From April 1, 2020 to May 20, 2020, 313 in-patients (≥14 years) of the University Medical Center Rostock were interviewed by questionnaires and 51 relatives by phone. Subjective psychological distress was assessed by a distress thermometer [0 (not at all)−100 (extreme)]. The study also investigated stressors due to VR, psychological distress in dependence on demographic or disease-related data, currently used communication channels and desired alternatives and support.

**Results:**

Relatives were more psychologically distressed by VR than in-patients (59 ± 34 vs. 38 ± 30, *p* = 0.002). Loss of direct physical contact and facial expressions/gestures resulted in the most distress. Psychological distress due to VR was independent of demographics and indicates small positive correlations with the severity of physical restriction and the general psychological distress of in-patients. The most frequent ways of communication were *via* phone and social media. Frequently requested alternatives for patients were other interlocutors and free phone/tablet use, for relatives visiting rooms with partitions.

**Conclusion:**

VRs are a stressor for patients and their relatives. The establishment of visiting rooms with partitions and the free use of phones/tablets could reduce the additional distress.

## Introduction

As social beings, humans depend on interactions with others in group bonds and relationships. Especially during states of exception as hospitalization social contacts and clear targeted communication are of great relevance; however, depending on the situation often limited.

Information exchange regarding disease and therapy between physician, patient, and relatives has been shown to influence patient satisfaction, treatment outcome, the healing process, and compliance (1–4). Contact with family members and close friends has positive effects on the health, everyday experience, and wellbeing of hospital in-patients (5). Relatives are not only supporters, but also affected persons and caregiver, which leads to a multiple burden (6). As a result of the knowledge of the importance and positive effects of the patient-relative relationship, hospitals have established visiting hours. Visitation restrictions (VR) have existed since the first hospitals were founded in the early 1800's. These were to reduce the spread of diseases and protect patients and their families from stress (7). In 2020, severe restrictions on visiting hours and bans on visiting occurred as a result of the COVID-19 pandemic. WHO and the European Center for Disease Prevention and Control published strict public health measures and guidelines to reduce the spread of the Coronavirus (8). The German Bundestag declared an “epidemic situation of national significance” in March 2020 (9) and enacted legal Corona protection measures based on the WHO guidelines. In the middle of March 2020, severe VRs in hospitals were determined (10). Based on the evidence that isolation/quarantine for the prevention of infectious diseases can cause mental health problems, such as depression, anxiety, and insomnia, there has been intense debate about VR (11, 12). The psychological impact of VR resulting from the COVID-19 pandemic on hospitalized patients and their families is largely unknown. Preliminary study results on investigations in vulnerable groups (nursing home residents, patients in palliative and intensive care units including neonatology), relatives of hospitalized children, and those who tested positive for COVID-19 and their relatives showed increased lonesomeness, depressive symptoms, agitation, aggression, decreased cognitive abilities, and general dissatisfaction for patients. For relatives, concerns, fears, and insecurities occurred (13–19). The present study aimed to investigate prospectively: (I) Whether hospitalized patients and their relatives experience different levels of psychological distress as a result of COVID-19-related VR? (II) Which items are particularly distressing? (III) Whether demographic and disease-related data provide information about psychological distress? and (IV) Which communication channels alternative to personal contact are currently used and which additions in terms of communication channels are desirable?

## Materials and Methods

### Study Design

The prospective study was designed as a two-arm cross-sectional study. A survey of in-patients and their relatives (relative was defined as the most important contact person) was conducted by questionnaire in person (patients) or by telephone interview (relatives).

### Patient-Sample, Inclusion Criteria

From April 1st until May 20th, 2020, a self-designed questionnaire survey of in-patients (age ≥14 years) was conducted at 17 somatic clinics of the University Medical Center Rostock (UMR) with various areas of care. Questionnaires were only handed out to patients once during their stay with a length of stay ≥ 2 days. Further inclusion criteria were: ability to consent, German-speaking, and physical and cognitive ability to complete a questionnaire. For underage patients (14–17 years), these criteria applied with regard to the legal guardians. The patient questionnaire was administered during the informed consent interview to minimize the number of contacts. Questionnaires were distributed and collected by medical staff, nursing staff, and study center staff.

### Survey of Relatives

Patients were asked to provide a relative with contact details. If a relative was named, the study center staff contacted that person by telephone. After consent was given, the interview was conducted according to a standardized interview template. The interviews had an approximate duration of 10 min.

Participation in the survey was voluntary, and all patient/relative data were analyzed in a pseudonymous manner. The study was reviewed and approved by the ethics committee of the University of Rostock (A2020-68).

### Visiting Restrictions/Exceptions

From March 13th, 2020, strict visiting restrictions to the in-patient areas of the Rostock University Medical Center applied. In individual cases, it was possible to deviate from this procedure. This resulted in inconsistent procedures for different areas. In wards with primarily cure-oriented intentions, patients with palliative diseases were under certain circumstances allowed to receive visitors. On the palliative ward, a maximum of two visitors per day were allowed to visit dying patients, only. Minor children were allowed to be accompanied by a healthy caregiver. The procedure in each individual case was determined by the facility manager of the respective department. The senior physicians in charge of the wards ensured implementation in consultation with the nursing teams. From May 20th, 2020, the strict visitation restrictions were abolished. Patients were then allowed to receive visits from caregivers again under strict conditions. This marked the end of this survey.

### Questionnaire/Interview

#### Demographics

Assessed were age, gender, living situation, and the patient-relative relationship (e.g., spouses).

#### Disease-Related Data

The following questions were asked of the patient: reason (diagnosis) for hospitalization, duration of illness to date, whether first hospitalization/in-patient stay, number of days spent as an in-patient, and expected length of stay.

Patients and relatives were asked to indicate on a distress thermometer (0 not at all and 100 extremely) how much they are currently physically restricted and under psychological pressure.

#### Importance of Communication

All participants were asked by means of 5-level Likert scales how important communication is in everyday life and direct communication with relatives, friends, etc. to them.

#### Attitude Toward Visitation Restrictions and Their Effects

Patients and relatives recorded: preferred frequency of visits, missed communication elements (e.g., facial expressions/gestures), understanding of the visitation restrictions, the general and personal perception of the VR on 5-point Likert scales, and the severity of the communication restriction. The strength of subjective psychological distress as a result of the VR was recorded using a distress thermometer (0 not at all−100 extremely). To be able to assess which proportion of the patients/relatives were distressed and to what extent, the following grouping was performed: Value “0” on the visual analog scale (VAS) = “not stressed,” VAS > 0 ≤ 30 = “slightly stressed,” VAS > 30 ≤ 70 = “moderately stressed,” VAS > 70 ≤ 90 = “highly stressed,” and VAS > 90 = “very highly stressed.”

#### Current and Desired Communication Channels

Patients and relatives were asked to provide information about the technologies used and ways of communication under the given conditions. In addition to given answer options, the respondents had the opportunity to add further technologies. Furthermore, wishes and possibilities for improvement in communication were surveyed. In addition to the predefined answer options, there was also the possibility of free-text options.

#### Statistics

In addition to descriptive analysis, interval-scaled data were tested for normal distribution using the Shapiro–Wilk test. Depending on the scale level, correlations and mean differences were tested using the Pearson chi-square test, Spearman correlation, and Mann–Whitney U test, respectively. The level of significance was set at *p* ≤ 0.05. Cramer's V (CV), effect size (ES), and correlation coefficient *r*, respectively, were used to interpret the strength of the relationships depending on the scale level. SPSS 22.0 (SPSS Inc., Chicago, IL, USA) was used for the statistical analysis of the data.

## Results

A total of 313 patients participated in the survey. These provided eligible 85 relatives, 51 of whom agreed to the interviews.

### Demographic Data

The questionnaires were completed by 120 (38%) women and 191 (61%) men (no sex *n* = 2). The mean age of the total cohort was 60 ± 16 years. Two hundred and seventeen (69%) of the patients lived with partner(s) and/or child(ren) at the time of hospitalization (**Table 2**).

Interviews were conducted with 51 relatives (40 (78%) women and 11 (22%) men) with an average age of 60 ± 13 years. Seventy-eight percent of the relatives were married to the patients (Table 1). The relatives-patients groups differed in gender distribution (*p* < 0.001, ES = 0.508).

**Table 1:** Patient- and relatives-characteristics, study results.

	**Variable**	***n*** **=** **x (%) or**		***p***
		**mean** **±SD (range)**		
		**Patients**	**Relatives**	
**Patient- and relatives-characteristics**	Total cohort	51	51	
	Sex			<0.001*
	Female	14 (28)	40 (78)	
	Male	37 (72)	11 (22)	
	Age [years]	61 ± 17 (15–89)	60 ±13 (33–82)	0.377
	Patient and relative living together			
	Yes	45 (88)		
	Patient and relative relationship			
	Married, cohabiting partner	40 (78)		
	Parent, child, other, N/A	11 (22)		
	First hospitalization			
	Yes	8 (16)		
	Days in hospital when interviewed			
	≤ 5	26 (51)		
	>5	23 (45)		
	N/A	2 (4)	
	Physical restriction (0 = none to 100 = extreme)	43 ± 29 (0–100)	16 ± 23 (0–80)	<0.001*
	General psychological distress (0 = none to 100 = extreme)	41 ± 28 (0–100)	54 ±30 (0–100)	0.043*
**Study results**	Psychological distress due to visitation restrictions (0 – none, 100 – extreme)	38 ±30 (0–100)	59 ± 34 (0–100)	0.002*
	Importance of communication in everyday life Very unimportant Unimportant Rather unimportant Important Very important	0 (0) 2 (4) 8 (16) 21 (41) 20 (39)	0 (0) 0 (0) 0 (0) 15 (29) 36 (71)	<0.001*
	Importance of direct communication in everyday life Very unimportant Unimportant Rather unimportant Important Very important	1 (2) 3 (6) 7 (14) 21 (41) 18 (35)	1 (2) 0 (0) 3 (6) 5 (10) 42 (82)	<0.001*
	Desired visit frequency <1 times per week 1–2 times per week Every 2–3 days Daily Several times a day	6 (12) 10 (20) 16 (31) 16 (31) 1 (2)	2 (4) 2 (4) 10 (20) 33 (65) 0 (0)	<0.001*
	Missing elements of communication			
	Direct physical contact	29 (57)	37 (73)	0.049*
	Facial expression and gestures	23 (45)	37 (73)	0.002*
	Voice	22 (43)	16 (31)	**0.280**
	Nothing	12 (24)	4 (8)	0.036*
	Current contact via			
	Visit (special regulation)	8 (16)		
	Phone	45 (88)		
	Text-only messages	9 (18)		
	Video calls	11 (22)		
	Social media	32 (63)		
	Desired support On mobile phone use For video calls Rooms/times for telephone calls Rooms/times for video calls Free bed phone Free use of tablet/pc Other interlocutors Others	6 (12) 3 (6) 9 (18) 6 (12) 12 (24) 8 (16) 19 (37) 3 (6)	1 (2) 3 (6) 8 (16) 6 (12) 2 (4) 6 (12) N/A 20 (40)^1^	

*SD, standard deviation; p, significance value; N/A, not available*.

Bold/* statistically significant values (p ≤ 0.05).

*^1^Visit in a visiting room (with partitions) was indicated 16 times, 1 time each help with the use of the tablet, use of a ward tablet, conversation with the physician, and the possibility to meet/see outside or from the balcony*.

### Diseases-Related Data

Information on disease-related data can be found in Table 2. One-quarter of the patients each were assigned to neurological, surgical, or internal medicine institutions. Patients in the palliative care unit and patients hospitalized for COVID-19 infection were grouped under “palli/infect” (8%). “Other” facilities (18%) included radiation therapy, psychosomatics, dermatology, and pediatric and adolescent clinics. Approximately one-third of the patients surveyed were in-patients due to oncological disease.

**Table 2:** Patient characteristics and association with psychological distress as a result of visit restriction^a^.

**Variable**	***n* = x (%) or**	***p***	**CV or r**
	**mean ±SD (range)**		
Total cohort	313		
Sex		0.193	CV = 0.141
Female	120 (38)		
Male	191 (61)		
Diverse, N/A	2 (1)		
Age [years]	60 ± 16 (15 – 89)	0.173	r = −0.078
Living situation		0.451	CV = 0.114
Alone	74 (24)		
With partner or child(ren)	217 (69)		
Nursing or retirement home, N/A	22 (7)		
Assignment of facilities		0.351	CV = 0.120
Neurology	71 (23)		
Surgery^1^	78 (25)		
Internal medicine^2^	81 (26)		
Palliative care / Infectology	25 (8)		
Others^3^	58 (18)		
Oncological disease		0.757	CV = 0.084
Yes	110 (35)		
No	162 (52)		
N/A	41 (13)		
Duration of illness [month]		0.637	CV = 0.102
<3	143 (46)		
3–12	78 (25)		
>12	76 (24)		
N/A	16 (5)		
First hospitalization			
Yes	71 (23)		
No	239 (76)	0.991	CV =0.031
N/A	3 (1)		
Days in hospital when interviewed		0.307	CV = 0.127
≤ 5	153 (49)		
> 5	150 (48)		
N/A	10 (3)		
Expected additional days in hospital (pts perspective)		0.363	CV = 0.119
≤ 7	165 (53)		
>7 or unknown	142 (45)		
Physical restriction (0 = none to 100 = extreme)	44 ± 29 (0–100)	<0.001*	r = 0.233
General psychological distress (0 = none to 100 = extreme)	38 ± 29 (0–100)	<0.001*	r = 0.458

^a^*On a scale from 0 (none) to 100 (extreme), the psychological distress resulting from the visit restriction is on average 40 ± 32*.

*SD, standard deviation; p, significance value; CV, Cramer's V; r, correlation coefficient; N/A, not available*.

*^1^General-, Visceral-, Vascular- and Transplant-Surgery, Otorhinolaryngology, Head and Neck Surgery, Urology, Neurosurgery, Trauma-, Hand- and Reconstructive-Surgery.*

*^2^Hematology, Oncology, Pneumology, Gastroenterology, Cardiology, Nephrology, Endocrinology.*

*^3^Radiation therapy, Psychosomatics, Dermatology, Children's Hospital.*

Bold/*statistically significant values (p ≤ 0.05).

Of all patients, physical impairment with a mean of 44 ± 29 was reported, and the current psychological distress with 38 ± 29.

The 51 family members reported a mean of 16 ± 23 for physical limitation and 54 ± 30 for current psychological distress. There were significant mean differences between patients and relatives for both factors (*p* < 0.001, ES = 0.466 and *p* = 0.043, ES = 0.200, respectively; Table 1).

### Importance of Communication

Daily communication was considered important to very important by 80% of the patients and 100% of the relatives (*p* < 0.001, ES = 0.360). Direct communication with relatives, friends, etc. was considered (very) important by 76% of patients and 92% of relatives (*p* < 0.001, ES = 0.442; Table 1).

### Attitude Toward Visitation Restrictions and Their Effects

Desired visit frequencies and missed communication elements are shown in Table 1. While 33% of the patients wanted daily visits, 65% of the relatives did (*p* < 0.001, ES = 0.345). Most frequently, both, patients and relatives, missed direct physical contact and nonverbal communication by means of facial expressions and gestures.

Comprehension of the VRs was 96% for each of the patients and relatives, respectively.

**Figure 1:**
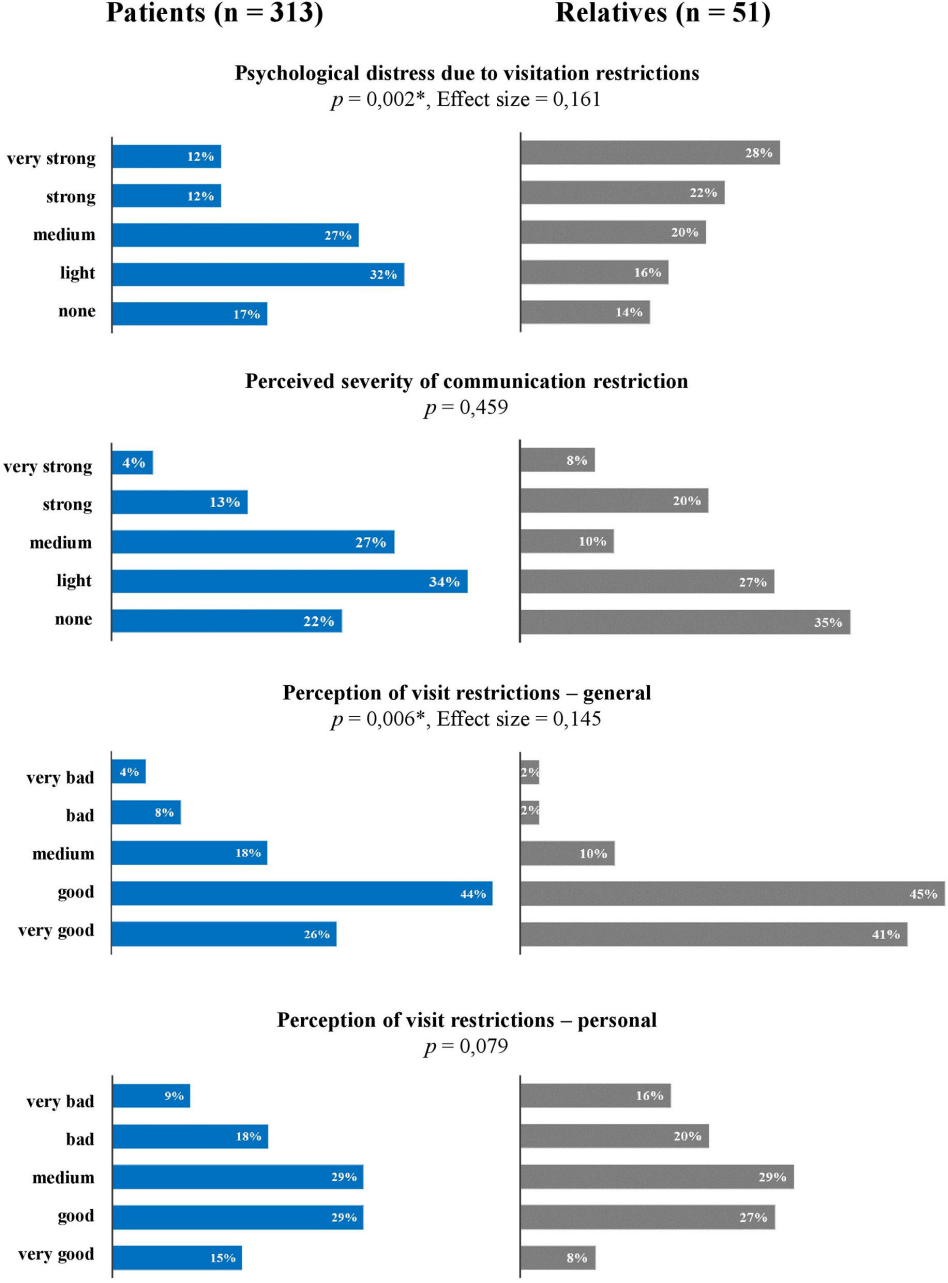
Psychological distress and perception of visit restrictions by patients and relatives. ^*^Statistically significant mean differences are indicated with *p < 0.05.

Figure 1 gives a graphical overview of the psychological distress and the perception of VR in patients compared to relatives. On average, patients reported psychological distress due to VR as 40 ± 32, whereas relatives reported it as 59 ± 34. The proportion of severely and very severely distressed was higher among relatives (*p* = 0.002). The sex-stratified analysis shows a higher psychological distress of the relatives in both genders compared to the patients (male: patients vs. relatives 40 ± 32 vs. 66 ±29, *p* = 0.012, ES = 0.179; female: patients vs. relatives 41 ± 33 vs. 56 ± 35, *p* = 0.014, ES = 0.196).

As shown in Table 2, there are no associations between the severity of psychological distress due to VR and demographic characteristics. In relation to the disease-related data, there were small or medium associations between psychological distress due to VR and the degree of physical impairment (*p* < 0.001) or general psychological distress (*p* < 0.001). These associations did not become apparent to relatives. The variance resolution between the parameters “general psychological distress” and “psychological distress due to VR” was 21% (patients) and 8% (relatives).

### Current and Desired Communication Channels

Of the 51 relatives, eight (16 %) had special visitation rights during the study period. The most frequently used means of communication were telephony and social media, which were used by 45 (88 %) and 32 (63 %) patient/relative pairs, respectively. Videotelephony was used to communicate by 11 (22%) patients/relatives pairs (Table 1).

Of all in-patients, 19 (37%) wished for other interlocutors (e.g., other in-patients and caregivers) as alternative visitors. Twelve (24%) wanted free bed phones and eight (16%) free use of tablet/PC. Relatives primarily used free text when indicating desired alternatives. Analysis of responses revealed that 16 (32%) desired patient visitations and suggested visitation rooms with partitions (e.g., glass partitions) as an option.

## Discussion

The study revealed that VR in hospitals to control the COVID-19 pandemic is an additional stressor for patients and their relatives. In the investigated cohort of in-patients and their relatives regardless of gender, relatives were more psychologically stressed by VR than patients. Direct physical contact and facial expressions/gestures were missed most by patients and relatives. Visitor rooms with partitions are a potential alternative to reduce psychological distress due to VR, especially for relatives. In the following, the causes and possible consequences of psychological distress due to VR and recommendations for action are discussed.

### Attitude Toward Visitation Restrictions and Their Effects

Almost all patients and relatives had an understanding of the VR and generally considered them to be (very) good. This is consequently considering the aim and reason for the VR was to protect these groups of people from infection, among other things. Nevertheless, the consequences of the VR had an impact on the mental conditions of patients and relatives. The collected data show that patients and relatives felt the psychological distress due to VR comparable to the general psychological distress they were exposed to in the actual situation. Both parameters, “general psychological distress” and “psychological distress due to VR” correlate only slightly with each other. The low variance resolution indicates that the VR is an additional stressor that is largely independent of other parameters. Consequently, VR places additional stress on the mental status of patients and relatives, as described by Meesters, among others, in mothers of infants (14).

Since the study presented here is cross-sectional, no concrete statements can be made about the medium- and long-term consequences among the respondents. In addition, the psychological distress due to VR was not assessed qualitatively. But based on the fact that elective procedures were severely restricted during the study period as part of the provision of ICU capacity in the context of the COVID-19 pandemic, it can be assumed that patients were confronted with more serious medical conditions (more than one-third were hospitalized due to cancer). The impact of VR during the COVID-19 pandemic on the wellbeing of hospitalized patients and their visitors, particularly in vulnerable populations, was examined by Inees et al. (20). Overall, the VRs were associated with negative emotions and detrimental effects on most in-patients and their families, especially in the context of end-of-life care (21). In end-of-life care, limiting visits or prohibiting visits resulted in inadequate emotional and spiritual care/support for patients and anxiety and despair among family members (22, 23). Patients were afraid of dying alone (24). In patients in the postoperative period, VR affected satisfaction with the hospital experience, and patients without visitors reported social isolation due to a lack of psychosocial support (25).

We suspect that the greater psychological distress on relatives results in part from the fact that they could not form their own impression of the patient's condition. It is known that inadequate information is a stressor for negative psychological effects such as posttraumatic stress symptoms, confusion, and anger (26). Furthermore, relatives feel helpless and guilty, because they cannot support their beloved ones (24). In addition, due to the extensive Corona protection measures, the relatives were exposed to additional restrictions (e.g., quarantine, contact blocks, and distance regulations) in everyday life, which have direct negative psychological consequences (11, 12, 26, 27). Furthermore, the data suggest that patients, although more physically limited than their relatives, felt cared for well in the hospital. Different data show that an in-patient environment with appropriate medical presence and participatory decision-making processes can contribute to anxiety reduction and higher satisfaction (2, 3, 28).

Even if VR contribute to a reduction of surgical site infections in postoperative patients (29), this does not outweigh the sum of the mentioned serious consequences for patients and their relative. Urgent action is needed to reduce or prevent the negative psychological effects and psychiatric symptoms resulting from VR.

### Impact of Demographic and Disease-Related Data

No predictors of the severity of psychological distress were identified within the demographic parameters analyzed. Also, length of hospitalization, single or repeated hospitalization, or cause of hospitalization (e.g., neurologic, surgical, and palliative) did not provide information on the severity of psychological distress due to VR. Only the severity of physical limitations and/or the general psychological distress the patients were under, showed a small positive correlation with psychological distress due to VR. For this reason, all patients should be given the necessary attention and offered help in dealing with VR.

### Used Communication Channels, Missed Items, and Desired Support

Relatives, independent of the gender, claimed to have more distress than in-patient due to VR. This can partly be explained by the higher importance of communication and interpersonal relationships among the relatives. Thus, the desire to visit the in-patients was more pronounced among the relatives than the desire to get visited among the patients. During the study period, patients and relatives communicated most frequently *via* phone and social media. Consequently, direct physical contact and facial expressions/gestures were missed the most. While patients mentioned other interlocutors (e.g., other patients and caregivers) as a possible alternative, the establishment of visiting rooms with protective measures (e.g., partition walls/glasses) was most frequently desired by relatives. Video calls were used by only a few and were also mentioned as an alternative by only a few. We suspect that lack of experience, technical difficulty, and lack of access to a device are barriers. However, unsuitability for patients, e.g., due to sedation, could also be a reason (30). According to the answers, especially patients could benefit from free phone, tablet, and/or PC use. In addition, the patients' and relatives' requests for rooms/times for phone and video calls indicate a desire for more privacy.

To protect mental health, the establishment of visiting rooms with partitions and free phone/chat rooms as alternative communication channels for patients and relatives in clinics should be examined and implemented. These measures could reduce psychological distress, especially for the relatives of in-patients, due to visual contact, an improved flow of information, and more privacy. Special attention should be paid to bed-ridden patients with limited communication skills (e.g., sedation, mechanical ventilation, and tracheostomy) and their relatives (31).

Since the Corona case numbers in the federal state, where the investigation was performed (Mecklenburg-Vorpommern) during the study period were rather low compared to other federal states (from mid-March to the end of May 2020, 15 Corona patients were treated at UMR, three of them intensively; all patients were discharged), it can be assumed that the psychological distress of patients and their relatives was even higher in risk areas. Further research is needed to take targeted measures to benefit the mental health of patients and relatives during pandemic periods.

### Limitations and Strengths

The survey was successfully conducted prospectively on a large cohort, despite the difficult baseline conditions, even for the study investigators. The service volumes and elective procedures were reduced at some hospitals during the survey period. The high number of oncological patients may be explained first by the fact that the study was led by the Department of Hematology, Oncology and Palliative Medicine (bias). Second, the treatments of these patients cannot be electively discharged. At the same time, this represents a strength, since specifically oncological patients were affected by VR.

Due to the involvement of a large number of clinics in the survey and the applicable contact restrictions, it was not possible to record exactly how many patients refused to participate, despite the coding of the questionnaires. A statement on the response rate is therefore not possible. As a result, a bias of the answers in the direction of socially desirable answers cannot be ruled out.

To keep the questionnaire and the interview duration short despite the complexity of the survey (aim: to increase the number of participants), only a visual analog scale and no standard psychological questionnaires were used to record psychological stress. As a consequence, no statements can be made about qualitative psychological stress. Whether and to what extent the visit restrictions had serious health consequences and which coping strategies were used should be investigated in further studies. Whether and to what extent the patients/relatives used psychotherapeutic services was not recorded and represents a further limitation of this study.

## Data Availability Statement

The raw data supporting the conclusions of this article will be made available by the authors, without undue reservation.

## Ethics Statement

The studies involving human participants were reviewed and approved by Ethics Committee of the University of Rostock. The patients consented to participate in the study by filling out the questionnaire. The relatives verbally agreed to be interviewed before the interview.

## Author Contributions

SF and CJ: conception of the work, data analysis and interpretation, and drafting the article. UK: conception of the work, data collection, and drafting the article. CSe: conception of the work and data collection. BK: data collection and head of study office. TH, FB, DS, CSc, and D-LD: data collection and critical revision of the article. All authors contributed to the article and approved the submitted version.

## Conflict of Interest

The authors declare that the research was conducted in the absence of any commercial or financial relationships that could be construed as a potential conflict of interest.

## Publisher's Note

All claims expressed in this article are solely those of the authors and do not necessarily represent those of their affiliated organizations, or those of the publisher, the editors and the reviewers. Any product that may be evaluated in this article, or claim that may be made by its manufacturer, is not guaranteed or endorsed by the publisher.
